# Inhibition of cardiomyocyte Sprouty1 protects from cardiac ischemia–reperfusion injury

**DOI:** 10.1007/s00395-018-0713-y

**Published:** 2019-01-11

**Authors:** Tarja Alakoski, Johanna Ulvila, Raisa Yrjölä, Laura Vainio, Johanna Magga, Zoltan Szabo, Jonathan D. Licht, Risto Kerkelä

**Affiliations:** 1 Research Unit of Biomedicine, Department of Pharmacology and Toxicology, University of Oulu, P. O. BOX 5000, 90014 Oulu, Finland; 2 University of Florida Health Cancer Center, Gainesville, FL 32610 USA; 3 Medical Research Center Oulu, Oulu University Hospital and University of Oulu, Oulu, Finland

**Keywords:** Ischemia–reperfusion injury, Myocardial infarction, Sprouty1, Extracellular signal-regulated kinase, Glycogen synthase kinase-3β

## Abstract

Sprouty1 (Spry1) is a negative modulator of receptor tyrosine kinase signaling, but its role in cardiomyocyte survival has not been elucidated. The aim of this study was to investigate the potential role of cardiomyocyte Spry1 in cardiac ischemia–reperfusion (I/R) injury. Infarct areas of mouse hearts showed an increase in Spry1 protein expression, which localized to cardiomyocytes. To investigate if cardiomyocyte Spry1 regulates I/R injury, 8-week-old inducible cardiomyocyte Spry1 knockout (Spry1 cKO) mice and control mice were subjected to cardiac I/R injury. Spry1 cKO mice showed reduction in release of cardiac troponin I and reduced infarct size after I/R injury compared to control mice. Similar to Spry1 knockdown in cardiomyocytes in vivo, RNAi-mediated Spry1 silencing in isolated cardiomyocytes improved cardiomyocyte survival following simulated ischemia injury. Mechanistically, Spry1 knockdown induced cardiomyocyte extracellular signal-regulated kinase (ERK) phosphorylation in healthy hearts and isolated cardiomyocytes, and enhanced ERK phosphorylation after I/R injury. Spry1-deficient cardiomyocytes showed better preserved mitochondrial membrane potential following ischemic injury and an increase in levels of phosphorylated ERK and phosphorylated glycogen synthase kinase-3β (GSK-3β) in mitochondria of hypoxic cardiomyocytes. Overexpression of constitutively active GSK-3β abrogated the protective effect of Spry1 knockdown. Moreover, pharmacological inhibition of GSK-3β protected wild-type cardiomyocytes from cell death, but did not further protect Spry1-silenced cardiomyocytes from hypoxia-induced injury. Cardiomyocyte Spry1 knockdown promotes ERK phosphorylation and offers protection from I/R injury. Our findings indicate that Spry1 is an important regulator of cardiomyocyte viability during ischemia–reperfusion injury.

**Electronic supplementary material:**

The online version of this article (10.1007/s00395-018-0713-y) contains supplementary material, which is available to authorized users.

## Introduction

Cardiovascular diseases are the major cause of mortality worldwide with coronary heart disease accounting to more than 40% of these deaths [13]. The standard of care for the prompt treatment of myocardial infarction (MI) is reperfusion therapies, including primary percutaneous coronary intervention and thrombolysis. A complete coronary occlusion lasting less than 20 min results in reversible injury and myocardial stunning [19, 22]. However, even after prolonged occlusion lasting 4–6 h, 30–50% of the area at risk remains viable. Studies utilizing various cardioprotective agents actually suggest that there is a therapeutic window to alleviate cardiac damage up to 24 h after ischemia [17]. Restoration of blood flow to ischemic myocardium limits infarct size and reduces mortality. The return of blood flow, on the other hand, can also result in additional cardiac damage, referred to as reperfusion injury [8]. Different approaches have been applied to activate cardioprotective signaling (e.g., ERKs and Akt) and to alleviate the ischemic injury to the myocardium (for review, see [16, 20, 43]). However, despite successful preclinical studies with various agents, such as insulin-like growth factor-1, cardiotrophin-1, and erythropoietin, none of these strategies have been sufficient to enter the clinics.

Activation of receptor tyrosine kinases by extracellular ligands (i.e., growth factors) induces phosphorylation of the cytoplasmic tail of the receptor and recruitment of adaptor and effector proteins to the receptor. Activated tyrosine kinase receptors signal to Ras via a protein complex formed by son of sevenless (Sos) and growth factor receptor-bound protein 2 (Grb2) to activate signaling via extracellular signal-regulated kinases (ERKs). ERK is inactivated by dephosphorylation by protein phosphatases such as protein phosphatase 2A and by dual-specificity phosphatase (DUSP)6 [41]. The tyrosine kinase receptor signaling to ERK pathway is also regulated by Sprouty (Spry) proteins, some of which prevent the recruitment of the Sos/Grb2 protein complex and, as a consequence, Ras activation is inhibited. There are four mammalian homologs (Spry1–4), of which all but Spry3 are expressed in the heart [35].

While the role of ERK in cardiomyocyte biology is rather well characterized, there is only little information of Spry proteins. Spry1 expression is increased in failing human hearts following therapy with a left ventricular assist device [23]. In an experimental model, cardiomyocyte-specific overexpression of Spry1 is not sufficient to reduce ERK activity or affect baseline cardiac phenotype [6]. However, the role of Spry1 in cardiomyocytes has not been investigated with loss of function approaches and no data exist on the role of cardiomyocyte Spry1 in regulating cardiomyocyte viability.

In this study, we investigated the role of Spry1 in cardiac ischemia–reperfusion (I/R) injury. We found that Spry1 levels are increased in mouse hearts following acute ischemic injury. We show that suppression of Spry1 in cardiomyocytes activates ERK and p38 mitogen-activated protein kinase (p38) signaling, attenuates cardiac troponin I (cTnI) release and reduces infarct size following I/R injury. Studies in isolated cardiomyocytes show that Spry1 knockdown reduces simulated ischemia-induced mitochondrial permeability transition pore opening and reveal a crucial role for glycogen synthase kinase- 3β (GSK-3β) in mediating the protective effect of Spry1 knockdown.

## Materials and methods (detailed methods in Online supplemental materials)

### Laboratory animals

All mice were of C57BL/6 background. They were maintained on a 12 h dark/light cycle (6 AM–6 PM) and housed in groups of ≤ 5 with unlimited access to water and chow. The experiments were performed with permission by the national Animal Experiment Board of Finland and by local authorities and conducted in accordance with the Directive 2010/63/EU of the European Parliament on the protection of animals used for scientific purposes. Spry^fl/fl^ mice, that have been previously published [3], have Lox-P sites flanking open reading frame (ORF) in exon 3 of the mouse Spry1 gene The mice, backcrossed for > 7 generations, were crossed with Myh6-Mer-Cre-Mer mice (Jackson laboratories) in the same background and also backcrossed > 7 generations. Mer-Cre-Mer is constitutively expressed but is inactive until treatment with tamoxifen. At 7 weeks of age, the mice were injected with tamoxifen (40 mg/kg i.p., Cat 13258, Cayman Chemicals, Ann Arbor, MI) on 2 consecutive days. Analysis for genomic DNA from heart samples of Spry1^fl/fl^ × Myh6-Mer-Cre-Mer mice showed efficient Cre-lox recombination in the myocardium upon tamoxifen treatment (Online Fig. 1). At 8 weeks of age, cardiomyocyte Spry1 knockout (Spry1 cKO) and control mice were subjected to cardiac I/R injury.

### Myocardial I/R model

Cardiac I/R injury was created by ligation of the left anterior descending artery (LAD) and releasing the slip knot after 30 min to allow for reperfusion of the myocardium as previously described in detail [12]. The mice were thereafter monitored for either 6 or 24 h and cardiac function and extent of cardiac injury (release of cTnI and infarct size or apoptosis) were analyzed. After the I/R procedure, mice were killed by cervical dislocation. Sham-operated mice were subjected to the same surgical procedure without ligation of the LAD. All operations were performed under isoflurane anesthesia (VetEquip vaporizer (VetEquip, Livermore, CA, USA), 2% isoflurane (Baxter, Deerfield, IL, USA), with 1 l/min flow of oxygen). Carprofen (Pfizer, New York, NY, USA) 5 mg/kg s.c. (injection volume 0.125–0.15 ml) was administered as pre-operation analgesia and buprenorphine (Orion Pharma, Espoo, Finland) 0.05 mg/kg s.c. (injection volume 0.1–0.3 ml) was administered as operation analgesia. Post-operation dehydration was prevented with s.c. injection of 5% glucose (injection volume 0.5–1.0 ml). All animals were monitored after the surgery and received a dose of buprenorphine (0.05 mg/kg) in the evening of operation day. Another dose of buprenorphine and a dose of carprofen (5 mg/kg) were administered the following morning.

### Cardiomyocyte hypoxia model

Neonatal rat ventricular cardiomyocytes (CMs) were subjected to hypoxia on the fourth day after siRNA transfections. For normoxia cells, DMEM (11966-025, Gibco, Dublin, Ireland) supplemented with 10 mM glucose was used. For hypoxia cells, DMEM supplemented with 10 mM deoxyglucose and 1 mM sodium dithionite (Fluka, Buchs, Switzerland) was used. During hypoxia, cells were incubated in C-Chamber (BioSpherix, New York, NY, USA), with oxygen level controlled at 0.1% with ProOx C21 (BioPherix, New York, NY, USA). When needed 3 µM GSK-3β inhibitor SB216763 (1616, Tocris Bioscience, Bristol, United Kingdom) was added to CMs 20 min before hypoxia experiment.

### Statistics

Statistical analysis was performed with IBM SPSS Statistics software (IBM, Armonk, NY, USA). Normally distributed data were analyzed with one-way ANOVA followed by Tukey’s post hoc test. When two groups were compared, Student’s *t* test was used. If normality was not achieved, data were analyzed with Mann–Whitney test, or in case of three or more groups with Kruskal–Wallis followed by Dunn’s post hoc test. Differences were considered statistically significant at the level of *P* < 0.05. Data are shown as mean ± SD.

## Results

### Spry1 protein levels are induced in ischemic cardiomyocytes in vivo

**Fig. 1:**
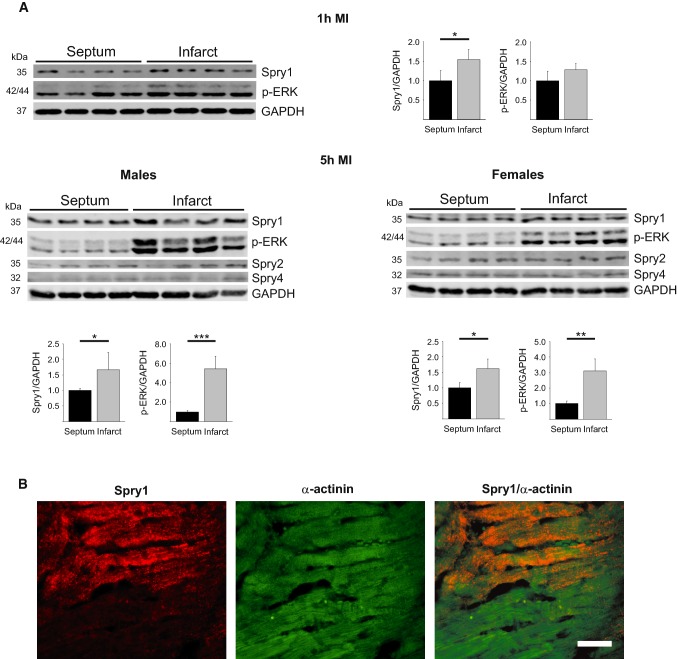
Analysis for Spry1 expression in ischemic myocardium. **a** Wild-type male mice were subjected to myocardial infarction (MI) for 1 and 5 h, and female mice for 5 h. Heart tissues from infarcted area and remote area (septum) were collected. Shown is immunoblot analysis of Spry1, phosphorylated extracellular signal-regulated kinase (p-ERK), Spry2, Spry4 and glyceraldehyde 3-phosphate dehydrogenase (GAPDH). Quantification of immunoblot analysis of Spry1 and p-ERK. GAPDH was used as a loading control. Data are shown as fold versus septum. *N* = 4–5; *P < 0.05, **P < 0.01, ***P < 0.001 versus septum. **b** Spry1^fl/fl^ mice were subjected to ischemia–reperfusion (I/R) injury and heart tissue was collected after 6 h of reperfusion. Shown is the immunofluorescence staining of formalin fixed and paraffin-embedded tissue sections from infarct area for Spry1 and cardiomyocyte marker α-actinin. Scale bar 40 µm

8-Week-old wild-type mice were subjected to experimental MI and protein samples from cardiac tissue were subjected to Western blot analysis. We found that MI induced an increase in levels of phosphorylated ERK and Spry1 protein in the infarcted areas of male myocardium compared to non-ischemic area (septum) that was more clear at 5 h after MI (Fig. 1a). Infarcted tissue of female mouse myocardium also showed induction in phosphorylated ERK and Spry1 expression analyzed 5 h after MI (Fig. 1a). On the other hand, myocardial infarction had no effect on expression of other cardiac Sprouty proteins, Spry2 or Spry4 (Fig. 1a).

We then investigated for Spry1 expression in resident cardiac cell types. In accordance with previous data [50], we found that Spry1 mRNA is expressed at eight-fold higher levels in fibroblast fraction compared to cardiomyocyte fraction in healthy hearts (Online Fig. 2). To assess if the increase in Spry1 protein expression occurs in cardiomyocytes in ischemic hearts, we subjected 8-week old male Spry1^fl/fl^ mice to I/R injury for 6 h and analyzed for localization of Spry1 expression by immunofluorescence microscopy. Analysis of cardiac sections showed that Spry1 is induced in ischemic areas in hearts of Spry1^fl/fl^ mice and colocalizes with cardiomyocyte marker α-actinin (Fig. 1b).

### Cardiomyocyte Spry1 knockdown protects from cardiac I/R injury

**Fig. 2:**
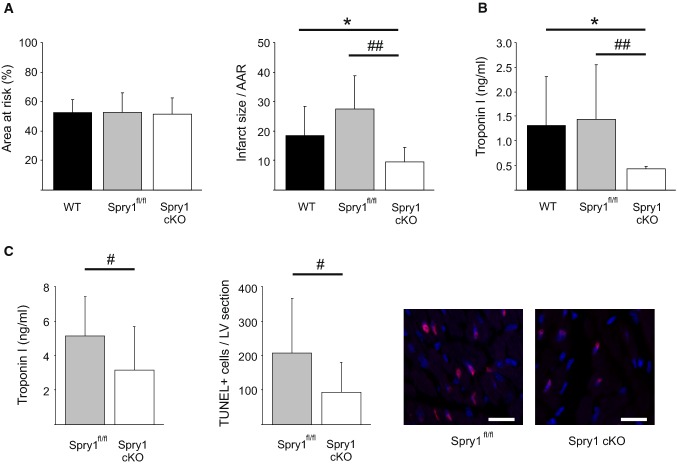
Spry1 cardiomyocyte knockdown protects from ischemia–reperfusion injury in vivo. Wild-type (WT), Spry1^fl/fl^ and Spry1 cardiomyocyte knockout (cKO) mice were subjected to 30 min of cardiac ischemia and 24 h of reperfusion, and subjected to analysis of infarct size, serum troponin I levels and analysis of cardiac function by echocardiography. **a** Analysis for area at risk (AAR) and infarct size relative to AAR derived from triphenyltetrazolium chloride (TTC) stainings. *N* = 9–13 per group. **b** Analysis for troponin I levels from serum samples. *N* = 9–13 per group. **c** Spry1^fl/fl^ and Spry1 cKO mice were subjected to 30 min of ischemia and 6 h of reperfusion, and subjected to analysis for serum troponin I levels and apoptosis by terminal deoxynucleotidyl transferase dUTP nick end labeling (TUNEL). Shown are also representative images of TUNEL labeling of sections from hearts of Spry1^fl/fl^ and Spry1 cKO mice. Scale bar 40 µm. *N* = 11–13 per group. *P < 0.05 versus WT mice and ^#^*P* < 0.05, ^##^*P* < 0.01 versus Spry1^fl/fl^ mice

To investigate for the role of cardiomyocyte Spry1 in regulation of cardiac ischemic injury, we utilized tamoxifen-inducible Cre-lox technique to delete Spry1 specifically from the cardiomyocytes. Wild-type (WT), Spry1^fl/fl^ and Spry1 cKO mice were subjected to cardiac I/R injury with 30 min of ischemia followed by 24 h of reperfusion. Analysis of cardiac structure and function by echocardiography prior to cardiac ischemia showed no difference between WT, Spry1^fl/fl^ and Spry1 cKO mice (Online Table 1). Analysis for the extent of cardiac injury at 24 h after I/R showed that Spry1 knockdown in cardiomyocytes had no effect on area at risk, but significantly reduced infarct size compared to both WT and Spry1^fl/fl^ mice (Fig. 2a). Analysis of plasma samples showed that Spry1 cKO mice had significantly lower plasma cTnI levels at the 24 h after I/R injury compared to both WT and Spry1^fl/fl^ mice (Fig. 2b). Echocardiography analysis showed significantly better preserved systolic function in Spry1 cKO mice compared to WT mice, while the difference between Spry1 cKO and Spry1^fl/fl^ mice did not reach statistical significance (Table 1). No difference was observed in left ventricular end-diastolic diameter between the groups, but the stroke volume was greater in Spry1 cKO mice compared to Spry1^fl/fl^ or WT mice (Table 1). Spry1 cKO mice also showed a 20% increase in cardiac output compared to control mice (Table 1).

**Table 1:** Echocardiography analysis of left ventricular structure and function following ischemia–reperfusion injury

	WT (*N* = 13)	Spry1^fl/fl^ (*N* = 10)	Spry1 cKO (*N* = 9)
LVID;d (mm)	3.98 ± 0.19	3.83 ± 0.17	3.97 ± 0.25
LVID;s (mm)	3.14 ± 0.29	2.92 ± 0.30*	2.84 ± 0.31*
LVPW;d (mm)	0.76 ± 0.09	0.76 ± 0.08	0.77 ± 0.08
LVPW;s (mm)	0.95 ± 0.08	0.99 ± 0.16	1.07 ± 0.19
LVVol;d (μl)	69.32 ± 7.99	63.21 ± 6.55	69.30 ± 10.68
LVVol;s (μl)	39.53 ± 8.76	33.07 ± 7.35	31.14 ± 8.36*
EF (%)	43.40 ± 8.19	47.73 ± 10.67	55.21 ± 9.02**
FS (%)	21.25 ± 4.77	23.86 ± 7.56	28.59 ± 6.08**
HR (BPM)	445 ± 32	459 ± 28	430 ± 39
SV (μl)	29.79 ± 4.74	30.14 ± 7.10	38.16 ± 7.60**^#^
CO (ml/min)	14.46 ± 2.91	15.03 ± 4.35	18.22 ± 4.80*
LVmass (mg)	105.49 ± 11.87	101.08 ± 9.05	110.58 ± 10.67
LVmass/BW (mg/g)	4.55 ± 0.84	4.16 ± 0.46	4.36 ± 0.66

Wild-type (WT), Spry1^fl/fl^ or Spry1 cardiomyocyte knockout (Spry1 cKO) mice were subjected to cardiac ischemia for 30 min followed by 24 h of reperfusion and cardiac structure and function were analyzed by echocardiography

Data are presented as mean ± SD; *P < 0.05, **P < 0.01 versus WT, ^#^*P *< 0.05 versus Spry1^fl/fl^

*LVID;d* Left ventricular internal dimension at end-diastole, *LVID;s* LV internal dimension at end-systole, *LVPW;d* LV end-diastolic posterior wall thickness, *LVPW;s* LV end-systolic posterior wall thickness, *LVVol;d* LV end-diastolic volume, *LVVol;s* LV end-systolic volume, *EF* ejection fraction, *FS* fractional shortening, HR heart rate, *SV* stroke volume, *CO* cardiac output, *LVmass* left ventricular mass, *LVmass/BW* LVmass versus body weight

Spry1^fl/fl^ mice and Spry1 cKO mice were then subjected to cardiac I/R injury for 6 h. Analysis of plasma samples showed significantly lower plasma cTnI levels in Spry cKO mice compared to the Spry1^fl/fl^ mice (Fig. 2c). Terminal deoxynucleotidyl transferase dUTP nick end labeling (TUNEL) staining of left ventricular (LV) sections showed a significant reduction in the number of apoptotic cells in ischemic areas of Spry1 cKO mouse hearts compared to the Spry1^fl/fl^ mouse hearts (Fig. 2c).

We next investigated if cardiomyocyte Spry1 deletion provides protection from I/R injury in female hearts. 8-Week-old female Spry1^fl/fl^ mice and Spry1 cKO mice were subjected to cardiac I/R injury for 24 h. Analysis for infarct size showed no difference in area at risk, but revealed a significant reduction in infarct size in female Spry1 cKO mice compared to female Spry1^fl/fl^ mice (Online Fig. 3a). Analysis of plasma samples of female mice showed a significant decrease in plasma cTnI levels in Spry cKO mice compared to the Spry1^fl/fl^ mice (Online Fig. 3b).

### Spry1 knockdown protects isolated cardiomyocytes from hypoxic injury

**Fig. 3:**
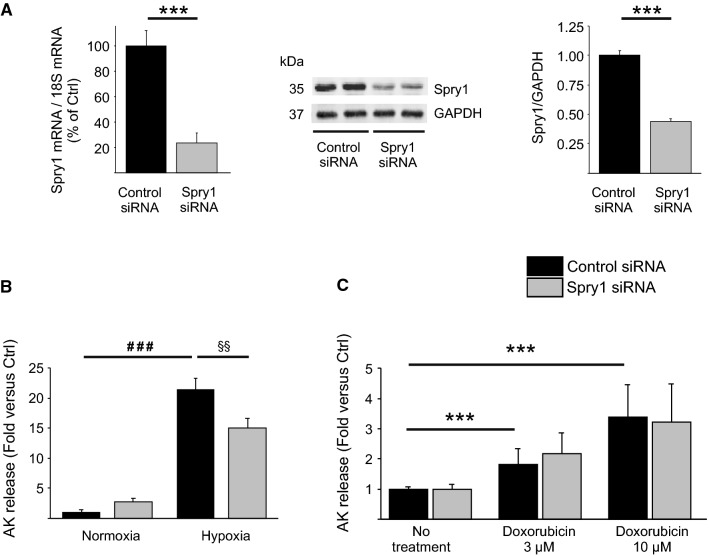
Spry1 knockdown in isolated cardiomyocytes protects from hypoxia–reperfusion injury. Neonatal rat ventricular cardiomyocytes (CMs) were transfected with Spry1 siRNA (100 nM) or control siRNA (100 nM). **a** Three days later, RNA samples were collected from CMs and analyzed by qPCR. Shown is the relative expression of Spry1 mRNA in Spry1 RNAi treated and control RNAi treated CMs. Results were normalized to expression of 18S ribosomal RNA (18S). *N* = 3 for each group. Four days later, protein samples were collected from CMs and analyzed by western blotting. Shown is immunoblot analysis for Spry1 and glyceraldehyde 3-phosphate dehydrogenase (GAPDH) and quantification of immunoblot analysis of Spry1. GAPDH was used as a loading control. Data are shown as fold versus control RNAi. *N* = 3–6. **b** RNAi-treated CMs were subjected to hypoxia. Shown is the analysis for released adenylate kinase (AK) from damaged CMs after 4 h of hypoxia. *N* = 4 for each group. **c** RNAi-treated CMs were treated with 3 μM and 10 μM doxorubicin for 24 h. Shown is the analysis for AK release from damaged CMs. *N* = 3 for each group. ***P < 0.001 versus control RNAi; ^###^*P* < 0.001 versus normoxia control RNAi and ^§§^*P* < 0.01 versus control RNAi in hypoxia

We then investigated if depletion of Spry1 in isolated cardiomyocytes offers protection from hypoxic injury. Spry1 RNAi in adult rat primary cardiomyocytes proved fruitless, since Spry1 mRNA expression was downregulated by 80–90% in non-treated healthy cardiomyocytes cultured for 2–4 days (Online Fig. 4). We then utilized RNAi in neonatal rat ventricular cardiomyocytes (CMs) which preserve their Spry1 expression in culture. Treatment of CMs with Spry1 siRNA led to 76% decrease in Spry1 mRNA and ~ 60% decrease in Spry1 protein levels (Fig. 3a). To investigate the effect of Spry1 RNAi on cardiomyocyte survival, Spry1 siRNA-treated CMs were subjected to simulated ischemia injury or cardiotoxic injury by doxorubicin treatment. Spry1-deficient cells showed markedly better survival [as assessed by release of adenylate kinase (AK) from ruptured cells] following 4 h of simulated ischemia (Fig. 3b). Spry1 knockdown, however, did not affect cardiomyocyte survival following treatment with 3 µM or 10 µM doxorubicin (Fig. 3c).

### Spry1 regulates ERK and p38 in cardiomyocytes

**Fig. 4:**
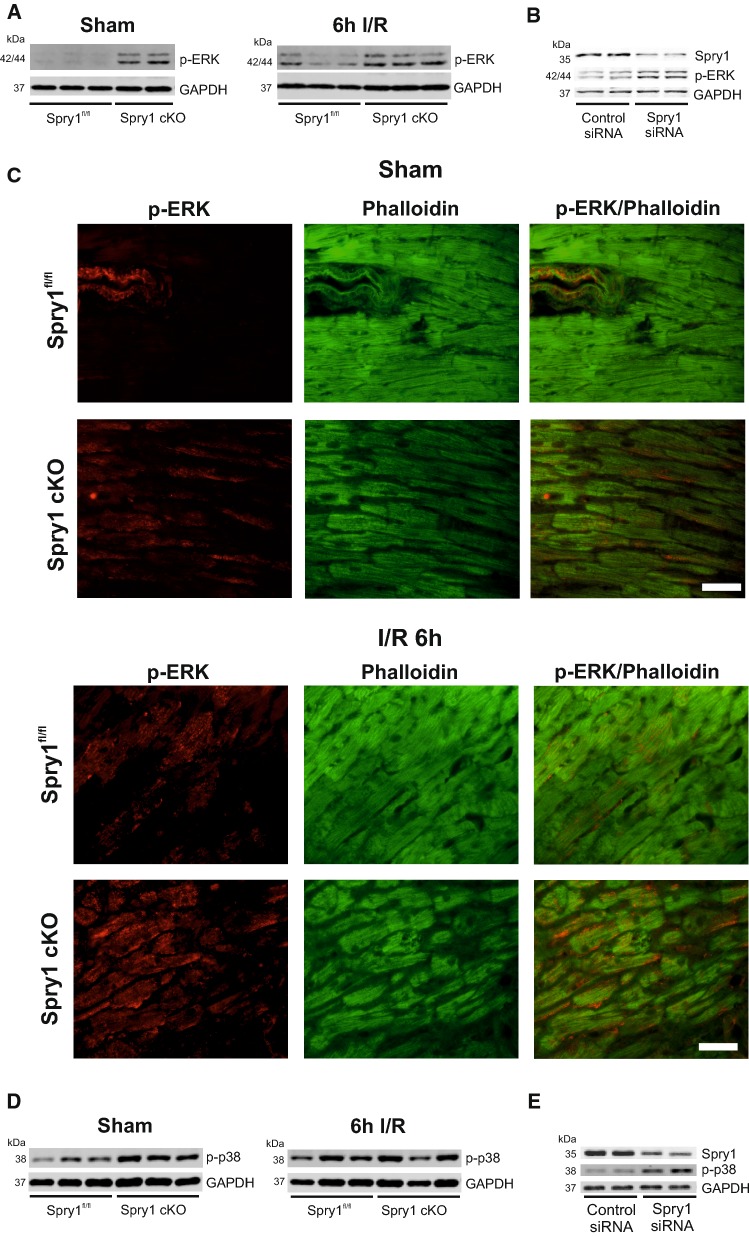
Spry1 regulates ERK and p38 in cardiomyocytes. Spry1^fl/fl^ and Spry1 cardiomyocyte knockout (cKO) mice were subjected to sham operation or to 30 min of ischemia (I/R), and heart tissue was collected after 6 h of reperfusion. **a** Immunoblot analysis of phosphorylated extracellular signal-regulated kinase (p-ERK) and glyceraldehyde 3-phosphate dehydrogenase (GAPDH) in sham and I/R operated Spry1^fl/fl^ and Spry1 cKO hearts. **b** Neonatal rat ventricular cardiomyocytes were transfected with Spry1 siRNA (100 nM) or control siRNA (100 nM). Four days later, protein samples were collected. Shown is the immunoblot analysis for Spry1, p-ERK and glyceraldehyde 3-phosphate dehydrogenase (GAPDH). **c** Immunofluorescence analysis for p-ERK and F-actin marker Phalloidin from control hearts and infarct areas from hearts subjected to 6 h of I/R injury. Scale bar 40 µm. **d** Immunoblot analysis of phosphorylated p38 (p-p38) and GAPDH in sham and I/R operated Spry1^fl/fl^ and Spry1 cKO hearts. **e** Immunoblot analysis for Spry1, p-p38 and GAPDH from protein samples of neonatal rat ventricular cardiomyocytes transfected with Spry1 siRNA (100 nM) or control siRNA (100 nM)

As Spry proteins have been shown to inhibit fibroblast growth factor induced Raf-ERK pathway activation [15], we assessed the effect of cardiomyocyte Spry1 knockdown on ERK activity. Western blot analysis for LV samples from healthy Spry1 cKO mice showed an increase in ERK phosphorylation in the myocardium compared to control Spry1^fl/fl^ mice (Fig. 4a, Online Fig. 5a). Analysis of ERK phosphorylation at 6 h after I/R injury showed that ERK phosphorylation remained elevated in ischemic myocardium of Spry1 cKO compared to ischemic areas of Spry1^fl/fl^ mouse hearts (Fig. 4a, Online Fig. 5a). In agreement with data from hearts in vivo, Spry1 knockdown in isolated cardiomyocytes increased ERK phosphorylation (Fig. 4b, Online Fig. 5b). Immunofluorescence analysis of cardiac sections showed that ERK is mainly phosphorylated in the vascular wall in hearts of sham-operated Spry1^fl/fl^ mice (Fig. 4c), whereas Spry1 cKO mice show ERK phosphorylation in cardiomyocytes (Fig. 4c). Immunofluorescence analysis after 6 h of I/R injury showed that ERK phosphorylation in cardiomyocytes is increased in both genotypes in ischemic areas, but Spry1 cKO cardiomyocytes show more intense staining (Fig. 4c). Spry1 thus negatively regulates ERK phosphorylation in cardiomyocytes at both baseline and following I/R injury.

Analysis of other central signaling pathways in cardiomyocytes showed that phosphorylation of p38 was increased in hearts of healthy Spry1 cKO mice compared to Spry^fl/fl^ mice (Fig. 4d, Online Fig. 5c). However, no difference was observed in p38 phosphorylation in ischemic tissues of Spry1^fl/fl^ and Spry1 cKO mice at 6 h after I/R injury (Fig. 4d, Online Fig. 5c). Analysis of other central cell survival pathways [phosphoinositide 3-kinase (PI3K)-Akt, Signal transducer and activator of transcription 3 (STAT3), c-Jun N-terminal kinase] showed no difference between hearts of Spry^fl/fl^ and Spry1 cKO mice either prior to ischemia or after the ischemia. In accordance with in vivo findings, Spry1 knockdown in isolated neonatal cardiomyocytes showed increased p38 phosphorylation (Fig. 4e, Online Fig. 5d). Spry1 thus regulates p38 phosphorylation in non-injured cardiomyocytes.

### Spry1 knockdown inhibits mitochondrial GSK-3β to protect from hypoxic injury

**Fig. 5:**
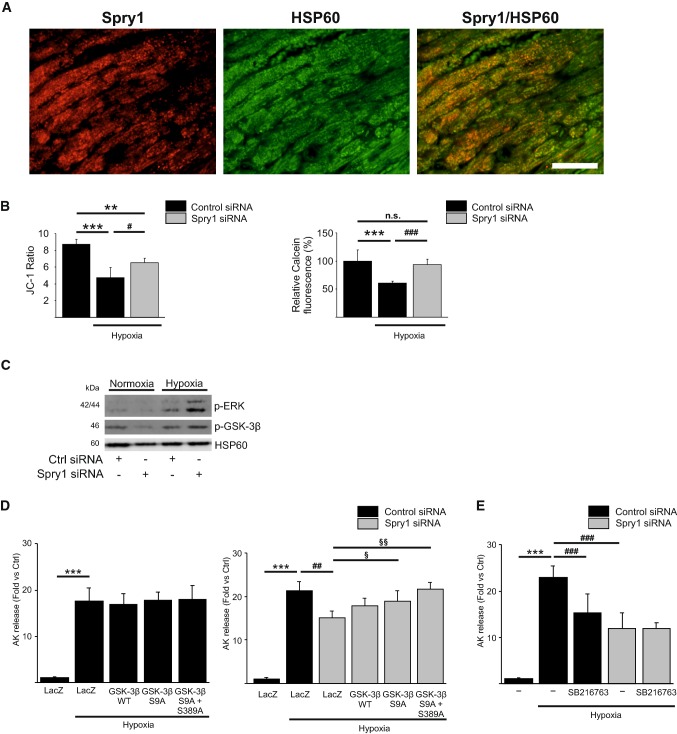
Glycogen synthase kinase-3β mediates the protective effect of Spry1 knockdown. **a** Spry1^fl/fl^ mice were subjected to 30 min of ischemia and heart tissue was collected after 6 h of reperfusion. Shown is the immunofluorescence staining of frozen sections from infarct area for Spry1 and mitochondrial marker heat shock protein 60 (HSP60). Scale bar 40 µm. **b** Neonatal rat ventricular cardiomyocytes (CMs) were transfected with Spry1 siRNA (100 nM) or control siRNA (100 nM), and 4 days later subjected to hypoxia for 4 h. Shown is the analysis for mitochondrial membrane potential expressed as ratio of JC-1 aggregate versus JC-1 monomer and detection of mitochondrial permeability transition pore opening using calcein cobalt assay expressed as relative Calcein-AM fluorescence (%). *N* = 4–8; **P < 0.01, ***P < 0.001 versus normoxia control RNAi, ^#^*P* < 0.05, ^###^*P* < 0.001 versus hypoxia control RNAi. **c** RNAi treated CMs were subjected to hypoxia for 4 h and mitochondrial protein samples were collected and analyzed by western blotting for phosphorylated extracellular signal-regulated kinase (p-ERK), phosphorylated glycogen synthase kinase-3β (p-GSK-3β) and HSP60. **d** RNAi-treated CMs were transduced with adenoviruses expressing LacZ, wild-type (WT) GSK-3β, Ser9Ala GSK-3β or Ser9Ala/Ser389Ala double mutant GSK-3β, and subjected to hypoxia for 4 h. Shown is the analysis for released adenylate kinase (AK) from damaged CMs after hypoxia. *N* = 4 for each group; ***P < 0.001 versus normoxia control RNAi, ^##^*P* < 0.01 versus hypoxia control RNAi and ^§^*P* < 0.05, ^§§^*P* < 0.01 versus hypoxia Spry1 RNAi. **e** RNAi-treated CMs were treated with GSK-3β inhibitor SB216763 (3 μM) for 20 min and subjected to hypoxia for 4 h. Shown is the analysis for released AK from damaged CMs after hypoxia. *N* = 4 each group; ***P < 0.001 versus normoxia control RNAi, ^###^*P* < 0.001 versus hypoxia control RNAi

Opening of the mitochondrial permeability transition pore (mPTP) at reperfusion is a critical determinant of I/R injury and inhibition of mPTP opening has been shown to reduce MI size and preserve cardiac function both in preclinical and clinical studies [5, 26]. To investigate if Spry1 regulates mPTP opening, we first investigated for Spry1 localization in injured cardiomyocytes. Analysis of LV sections from Spry1^fl/fl^ mice subjected to cardiac I/R injury for 6 h showed co-staining of Spry1 and mitochondrial marker heat shock protein 60 (HSP60) in cardiomyocytes (Fig. 5a). To determine if Spry1 regulates mitochondrial membrane potential (ΔΨm), Spry1 and control siRNA treated cardiomyocytes were subjected to 4 h of hypoxia and ΔΨm was measured with JC-1. We found that Spry1-deficient cardiomyocytes have significantly better preserved mitochondrial membrane potential compared to control cardiomyocytes (Fig. 5b). Similarly, analysis of mPTP opening by calcein cobalt assay showed better preserved mitochondrial membrane integrity in Spry1-deficient cardiomyocytes (Fig. 5b). Inhibition of GSK-3β has been shown to reduce mitochondrial membrane permeability transition and cell death, and ERK and p38 signaling pathways have both been shown to phosphorylate and inhibit GSK-3β [10, 25, 34, 49]. We then performed western blot analyses of pooled mitochondrial fractions from cardiomyocytes subjected to hypoxia and found that the levels of phosphorylated ERK and phosphorylated (inactive) GSK-3β were increased in mitochondria of Spry1-deficient cardiomyocytes compared to control siRNA transfected cardiomyocytes (Fig. 5c). To assess for possible role of GSK-3β in Spry1-mediated response, we transduced isolated cardiomyocytes with adenoviruses encoding for wild-type GSK-3β, GSK-3β harboring a Ser9Ala mutation and GSK-3β harboring Ser9Ala and Ser389Ala mutations. Phosphorylation of GSK-3β at Ser9 or at Ser389 has previously been shown to inactivate the kinase [48, 49]. We found that the forced activation of GSK-3β has no effect on hypoxia-induced cell death in control siRNA-treated cardiomyocytes (Fig. 5d). However, overexpression of GSK-3β (Ser9Ala) partially reverses the protective effect of Spry1 knockdown in hypoxic cardiomyocytes and overexpression of GSK-3β (Ser9Ala/Ser389Ala) completely abolishes the protective effect of Spry1 knockdown (Fig. 5d). Finally, we investigated if control and Spry1 siRNA-treated cardiomyocytes were sensitive to GSK-3β inhibition during hypoxia. We found that the treatment of control cardiomyocytes with SB216763 (3 μM) significantly reduces hypoxia-induced cardiomyocyte death (Fig. 5e). However, pharmacological inhibition of GSK-3β does not further protect Spry1-deficient cardiomyocytes from hypoxia-induced injury (Fig. 5e). These data thus indicate a crucial role for GSK-3β in regulating the protective response to Spry1 knockdown.

## Discussion

Despite an increasing number of patients suffering from ischemic heart disease, there are no pharmacological therapies in clinical use for treatment of cardiac I/R injury. Activation of RISK and Safe pathways (i.e., ERK, STAT and PI3K-Akt) by various growth factors and pharmacological agents have shown beneficial effects in attenuating ischemic cardiomyocyte injury in experimental models [18, 20]. However, this has not led to development of novel therapies. One important caveat with these approaches is that even with continuous growth factor stimulation, the activity of pro-survival pathways in cardiomyocytes is usually transient [47]. Growth factor-induced activation of ERK, for example, is abrogated in response to inhibitory feedback signaling to Ras and actually to nearly all ERK cascade components, by downstream kinases as well as by induction of inhibitors of tyrosine kinase signaling, such as Sprouty [27, 28]. In the context of G protein-coupled receptor (GPCR) activation, activated GPCRs are desensitized by phosphorylation by members of the family of G protein-coupled receptor kinases (GRKs) [11].

In the current study, we found that ischemia resulted in accumulation of Spry1 protein in cardiomyocytes in ischemic myocardium, but had no effect on expression of other Spry proteins in the myocardium. We then utilized cardiomyocyte Spry1 knockdown both in vivo and in vitro to investigate the role of Spry1 in cardiac I/R injury. We found that cardiomyocyte Spry1 knockdown in vivo reduced infarct size, reduced troponin I release and reduced the number of TUNEL positive cells in the myocardium following I/R injury. Data from in vitro studies showed that Spry1 knockdown was sufficient to reduce cardiomyocyte death following experimental hypoxia. Analysis of downstream signaling pathways regulated by Spry1 showed that Spry1 knockdown results in activation of ERK and p38 pathways in cardiomyocytes. Data from previous studies indicate that the key mechanism by which Spry proteins modulate cell proliferation and survival is inhibition of ERK pathway [31]. On the other hand, inhibition of Spry proteins in various cell types has been shown to induce robust ERK activation that varies in strength and duration [33]. ERK phosphorylation in the myocardium is induced following experimental ischemia and in human hearts in response to cardioplegia/reperfusion [21, 42]. Interestingly, both induction of ERK signaling and hypoxia have been shown to induce Spry1 expression [1, 40]. The noted increase in Spry1 levels in cardiomyocytes after ischemic injury may thus serve as a feedback mechanism to limit ERK activation. The role of the ERK cascade in regulating cardiomyocyte survival is well characterized. Inhibition of ERKs has been shown to exaggerate I/R-induced apoptosis and, conversely, increased ERK phosphorylation/activation is associated with protection from cardiac I/R injury [7, 29, 51]. However, the downstream mechanisms mediating the protective effects of ERK are not fully understood [42]. We found that Spry1 depletion in mouse cardiomyocytes augmented ERK phosphorylation after I/R injury and, notably, induced ERK activation at baseline, indicating that cardiomyocyte Spry1 regulates ERK in physiological conditions. Spry1 thus appears to be a central regulator of ERK in cardiomyocytes and, differently from ligand-induced ERK activation, Spry1 depletion results in persistent ERK phosphorylation that is preferable for a cardioprotective agent.

In addition to ERKs, we found that depletion of Spry1 in cardiomyocytes in vivo and in vitro resulted in increased p38 phosphorylation. Previously, activation of p38 signaling has only been observed in triple Spry1/Spry2/Spry4 knockouts in mouse embryonic fibroblasts [46]. p38 appears to have a dual role in myocardial I/R injury. p38 is activated rapidly in response to myocardial ischemia [30] and there are data indicating that inhibition of p38 during ischemia and/or reperfusion is cardioprotective [9, 20]. However, a clinical trial with losmapimod, an oral nonisoform selective inhibitor of p38, found no effect on necrosis biomarkers creatine kinase and TnI in patients with non-ST segment elevation acute MI [39]. On the other hand, the activity of p38 is increased during ischemic preconditioning and its inhibition abrogates the cardioprotection [36–38, 45]. p38 activation during preconditioning in vivo has actually been shown to attenuate p38 activation during ischemia [44]. In the current study, we found that Spry1 depletion both in vivo and in vitro induced phosphorylation of p38. However, cardiomyocyte Spry1 knockdown had no effect on p38 phosphorylation after I/R injury.

Opening of the mPTP has been shown to play a crucial role in cardiac I/R injury and ischemic postconditioning attenuating the mitochondrial permeability transition [2, 26]. Interestingly, there is evidence from NIH3T3 cells that Spry2 localizes in mitochondrial membranes [32]. In the current study, we found that the induction in Spry1 expression in cardiomyocytes after I/R injury was localized to cardiomyocyte mitochondria and Spry1 inhibition attenuated mitochondrial membrane permeability transition. Furthermore, Spry1 depletion did not protect from doxorubicin-induced cardiomyocyte death, which is triggered by mechanisms other than mitochondrial membrane permeability transition, such as through mitochondrial iron accumulation and topoisomerase-IIβ. While cyclophilin D is the best-characterized regulator of the mPTP, prior studies have also indicated a role for GSK-3β in regulating mPTP opening [14]. Genetic inactivation of GSK-3β has been shown to protect from I/R injury, whereas forced activation of GSK-3β exacerbated myocardial I/R injury [52]. Multiple signaling pathways have been shown to converge at the level of GSK-3β resulting in increased phosphorylation and inhibition of the kinase [4, 24, 25]. ERK has been shown to phosphorylate GSK-3β at the Thr43 residue priming GSK-3β for its subsequent phosphorylation at Ser9, resulting in inactivation of GSK-3β [10]. GSK-3β is also phosphorylated and inactivated at Ser389 by p38 [49], but the significance of this phosphorylation in cardiomyocytes is not known. We found that mitochondrial GSK-3β was Ser9 phosphorylated (i.e., inhibited) in Spry1-deficient cardiomyocytes following hypoxia. We then investigated if GSK-3β played a role in protective response to Spry1 knockdown and found that overexpression of constitutively active GSK-3β (Ser9Ala) modestly attenuated the protective effect of Spry1 knockdown. However, overexpression of GSK-3β also harboring a p38 target site mutation (Ser9Ala/Ser389Ala) fully abrogated the protective effect of Spry1 inhibition. On the other hand, overexpression of active forms of GSK-3β did not increase cell death in control cardiomyocytes subjected to hypoxia. In accord with the actions of active GSK-3β, pharmacological inhibition of GSK-3β did not further protect Spry1-deficient cardiomyocytes from hypoxic injury, while GSK-3β inhibition significantly reduced hypoxia-induced cell death in control cardiomyocytes. These data thus indicate that in the context of I/R injury, mitochondrial GSK-3β is a central downstream target of Spry1. However, given the robust activation of ERK and p38 upon Spry1 depletion, other mechanisms may also play a role in mediating the cardioprotective effect.

In summary, we found that myocardial ischemia promotes Spry1 protein expression in ischemic cardiomyocytes. Our experimental data show that cardiomyocyte Spry1 knockdown enhances ERK phosphorylation and protects from cardiac I/R injury. Data from the studies in isolated cardiomyocytes show that Spry1 knockdown protects from hypoxia-induced mitochondrial permeability transition pore opening and indicates a central role for GSK-3β in mediating the protective effect of Spry1 knockdown. Further studies are needed to evaluate if inhibition of Spry1 offers novel therapeutic strategy to alleviate cardiac ischemia–reperfusion injury.

## Electronic supplementary material

Below is the link to the electronic supplementary material.

Supplementary material 1 (PDF 1458 kb)

Supplementary material 2 (PDF 366 kb)
