# Supplementary material for: Inhibition of cardiomyocyte Sprouty1 protects from cardiac ischemia–reperfusion injury
Source: Basic Res Cardiol. 2019 Jan 11;114(2):7. doi: 10.1007/s00395-018-0713-y (PMC6329741; doi:10.1007/s00395-018-0713-y)
Supplement: Supplementary file 1 — Supplementary material 1 (PDF 1458 kb) [file 395_2018_713_MOESM1_ESM.pdf]

Inhibition of Cardiomyocyte Sprouty1 Protects From Cardiac Ischemia/Reperfusion Injury

Tarja Alakoski[1], Johanna Ulvila[1], Raisa Yrjölä[1], Laura Vainio[1], Johanna Magga[1], Zoltan Szabo[1], Jonathan D. Licht[2] and Risto Kerkelä[1,3]

[1]Research Unit of Biomedicine, Department of Pharmacology and Toxicology, University of Oulu, Finland

[2]University of Florida Health Cancer Center, Gainesville, FL 32610, USA.

[3]Medical Research Center Oulu, Oulu University Hospital and University of Oulu, Oulu, Finland

Address for correspondence:
Prof. Risto Kerkelä, MD, PhD
Research Unit of Biomedicine
University of Oulu
P.O.BOX 5000
FI-90014, Oulu, Finland
E-mail: Risto.Kerkela@oulu.fi
Tel. +358-50 350 3989

Online Figures

Online Fig. 1

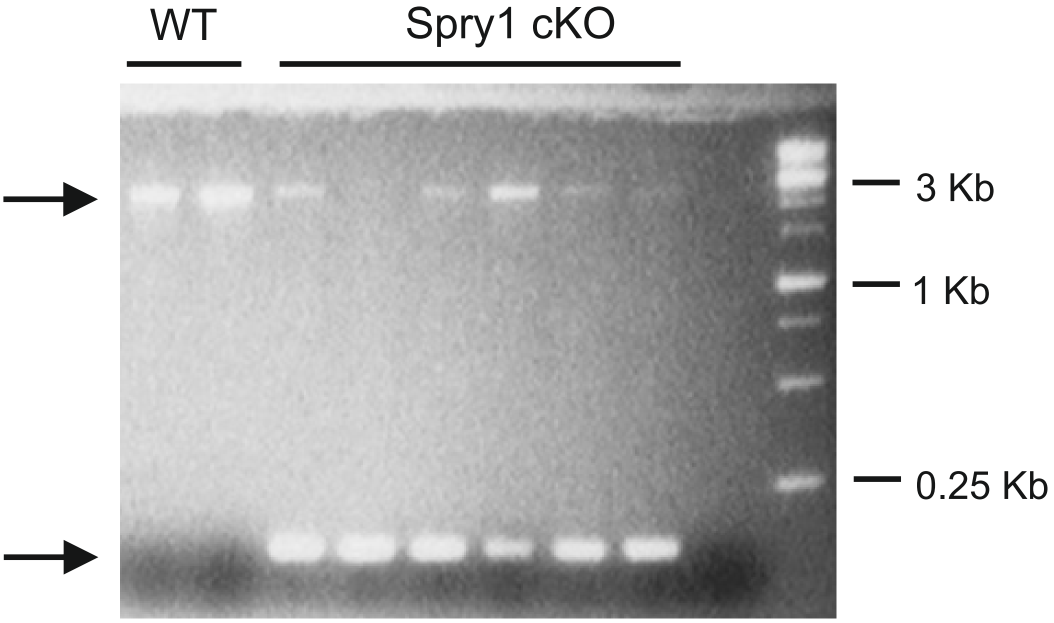

Online Fig. 1 Cre-lox recombination in MYH6-Mer-Cre-Mer x Spry1$^{fl/fl}$ (+tamoxifen) mice. DNA samples from hearts of wild type (WT) and Spry1$^{fl/fl}$ × Myh6-Mer-Cre-Mer mice treated with tamoxifen (Spry1 cKO) were genotyped using P2 and P3 primers as described in the Methods. DNA fragment size for intact Spry1 allele is 3 kb, and the size for Spry1 allele after Cre-mediated excision is 0.15 kb.

Online Fig. 2

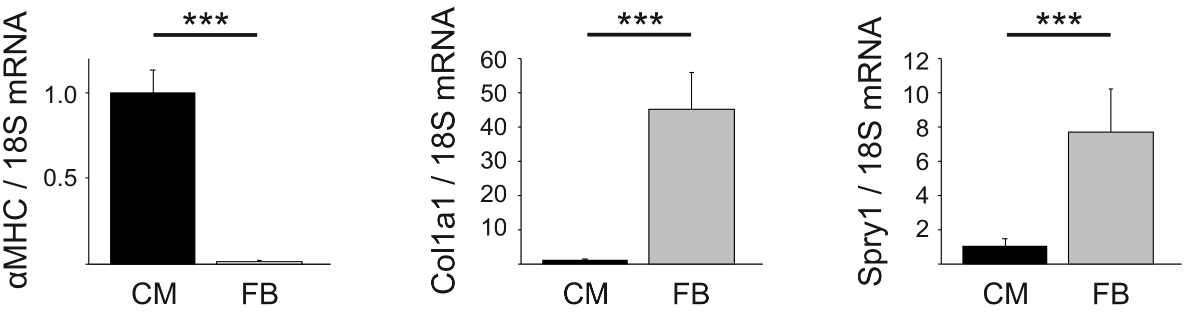

Online Fig. 2 Analysis for Spry1 expression in resident cardiac cells
qPCR analysis of the expression of collagen type I alpha 1 chain (Col1a1), and α myosin heavy chain (α-MHC) mRNAs in pools of fractionated mice cardiac cells. Col1a1 and α-MHC were used as markers for fibroblasts (FB), and cardiomyocytes (CM), respectively. Also shown is expression of Spry1 mRNA in FB fraction relative to that in the CM fraction. Results were normalized to expression of 18S ribosomal RNA (18S). N=5 for each group; ***P<0.001 versus CM.

Online Fig. 3

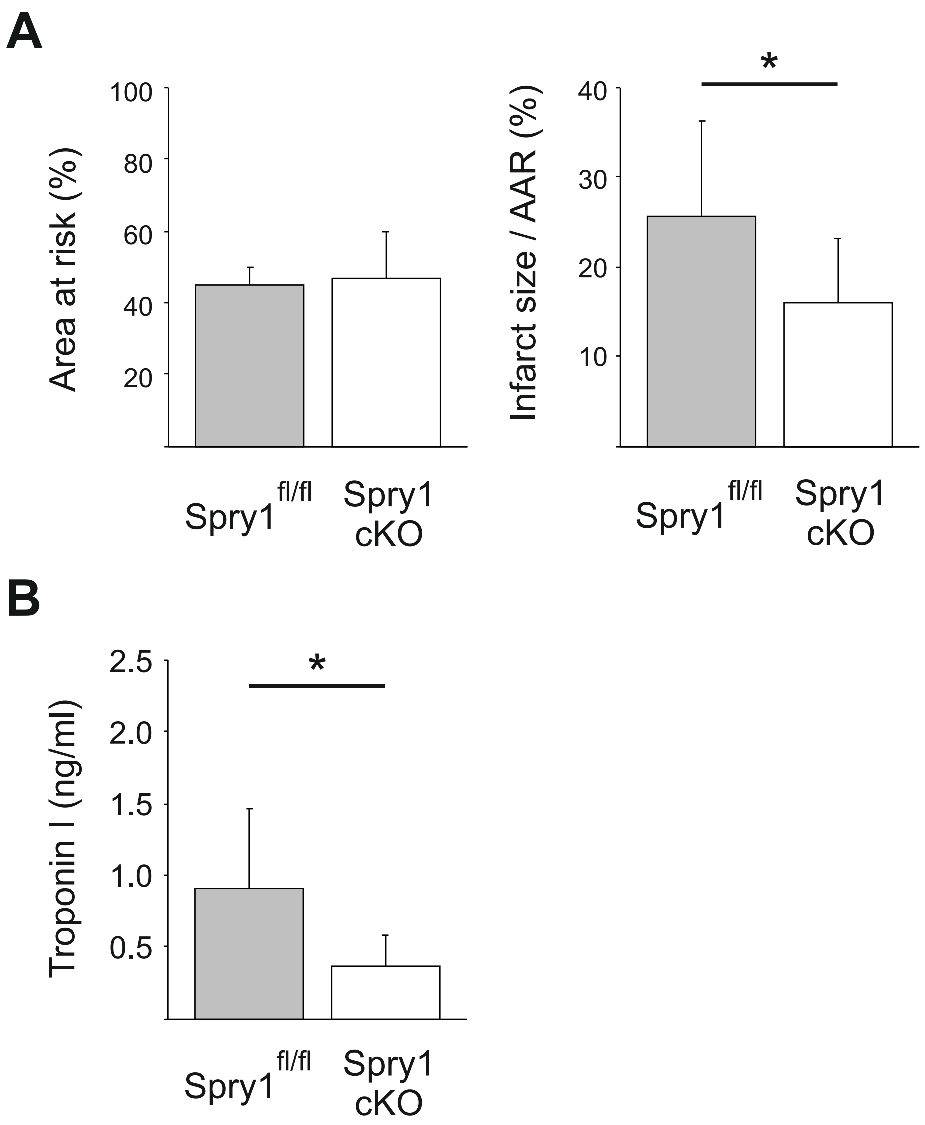

Online Fig. 3 Spry1 knockdown in female mice protects from ischemia-reperfusion injury
Female Spry1$^{fl/fl}$ and Spry1 cardiomyocyte knockdown (Spry1 cKO) mice were subjected to 30 minutes of ischemia and 24 hours of reperfusion, and analyzed for infarct size and serum troponin I levels. **a** Analysis for area at risk (AAR) and infarct size relative to AAR derived from triphenyltetrazolium chloride (TTC) stainings. **b** Analysis for troponin I levels from serum samples. N=7-8. *$P<0.05$ versus Spry1$^{fl/fl}$ mice.

Online Fig. 4

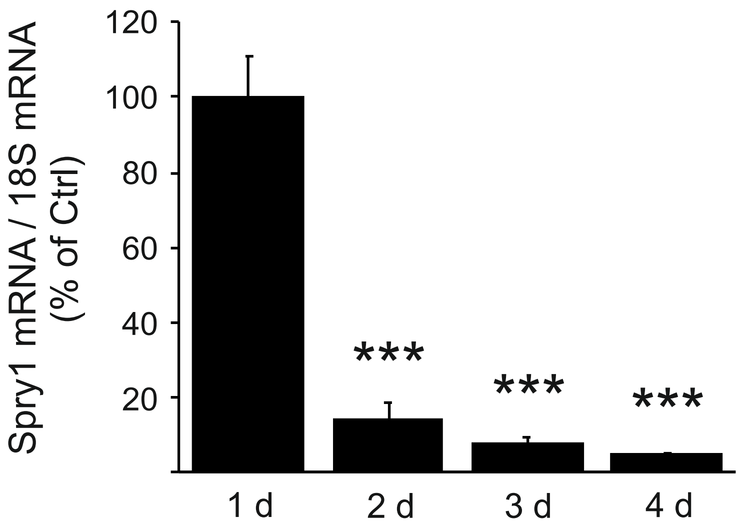

Online Fig. 4 Analysis for Spry1 expression in adult rat ventricular cardiomyocytes
Adult rat ventricular cardiomyocytes (ARVMs) were cultured for 1 to 4 days and RNA samples were collected. Shown is qPCR analysis of the expression of Spry in ARVMs. Results were normalized to expression of 18S ribosomal RNA (18S). N=3 for each group; ***$P<0.001$ versus Spry1 expression at day 1.

Online Fig. 5

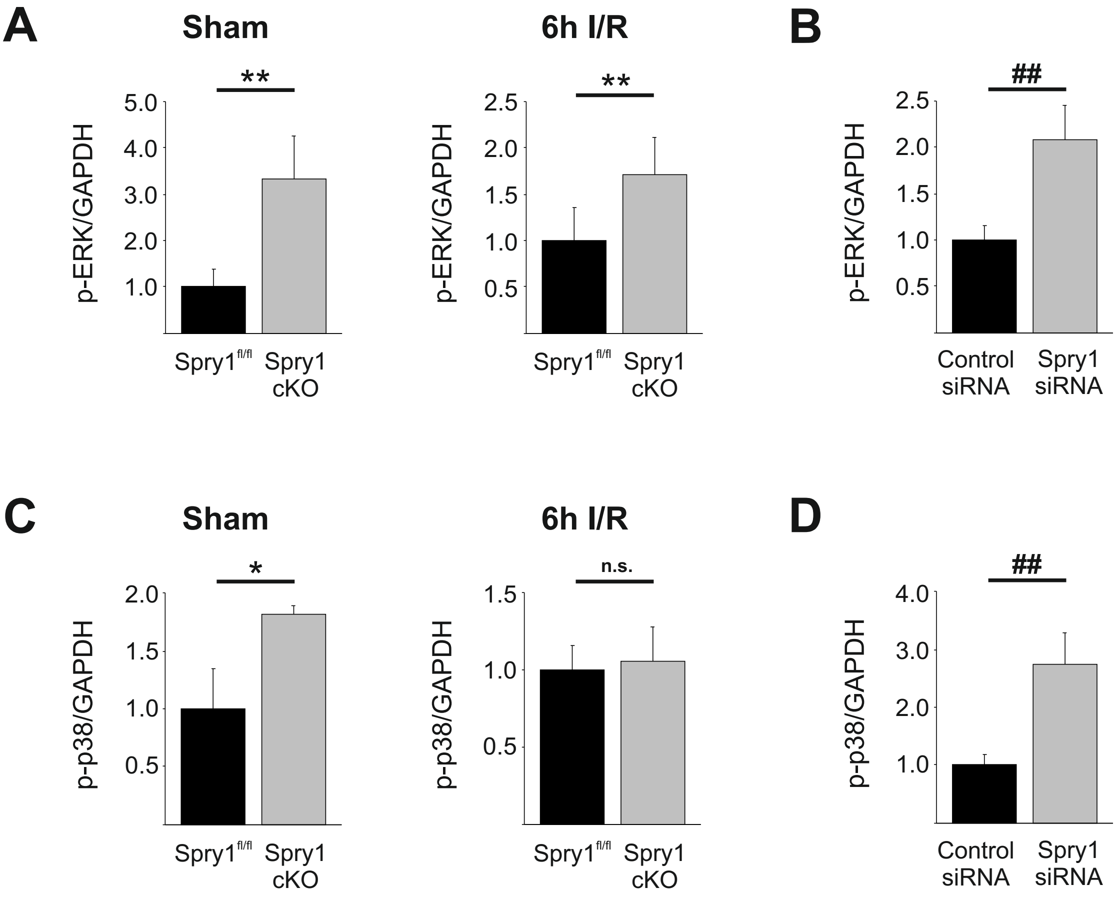

Online Fig. 5 Spry1 regulates ERK and p38 in cardiomyocytes
a and c Spry1$^{fl/fl}$ and Spry1 cardiomyocyte knockout (cKO) mice were subjected to sham operation or to 30 minutes of ischemia (I/R), and heart tissue was collected after 6h of reperfusion. Shown is quantification of Western blot analysis of phosphorylated extracellular signal-regulated kinase (p-ERK) and phosphorylated p38 (p-p38). Glyceraldehyde 3-phosphate dehydrogenase (GAPDH) was used as a loading control. Data are shown as fold vs Spry1$^{fl/fl}$. N=3-6; *P<0.05, **P<0.01 versus Spry1$^{fl/fl}$ mice. **b and d** Neonatal rat ventricular cardiomyocytes were transfected with Spry1 siRNA (100 nM) or control siRNA (100 nM). Four days later protein samples were collected. Shown is quantification of Western blot analysis of p-ERK and p-p38. GAPDH was used as a loading control. Data are shown as fold versus control siRNA. N=3-4; ##P<0.01 versus control siRNA.
