# Supplementary material for: Inhibition of cardiomyocyte Sprouty1 protects from cardiac ischemia–reperfusion injury
Source: Basic Res Cardiol. 2019 Jan 11;114(2):7. doi: 10.1007/s00395-018-0713-y (PMC6329741; doi:10.1007/s00395-018-0713-y)
Supplement: Supplementary file 2 — Supplementary material 2 (PDF 366 kb) [file 395_2018_713_MOESM2_ESM.pdf]

**Inhibition of Cardiomyocyte Sprouty1 Protects From Cardiac Ischemia/Reperfusion Injury**

Tarja Alakoski[1], Johanna Ulvila[1], Raisa Yrjölä[1], Laura Vainio[1], Johanna Magga[1], Zoltan Szabo[1], Jonathan D. Licht[2] and Risto Kerkelä[1,3]

[1]Research Unit of Biomedicine, Department of Pharmacology and Toxicology, University of Oulu, Finland

[2]University of Florida Health Cancer Center, Gainesville, FL 32610, USA.

[3]Medical Research Center Oulu, Oulu University Hospital and University of Oulu, Oulu, Finland

**Address for correspondence:**
Prof. Risto Kerkelä, MD, PhD
Research Unit of Biomedicine
University of Oulu
P.O.BOX 5000
FI-90014, Oulu, Finland
E-mail: Risto.Kerkela@oulu.fi
Tel. +358-50 350 3989

**Online Supplementary Materials**

## Supplementary Materials and methods

### Echocardiography

Echocardiography was performed by transthoracic echocardiography using Vevo 2100 high-frequency, high-resolution ultrasound system with a 40-MHz linear transducer (MS-550S, Visual Sonics, Toronto, Canada) under isoflurane anesthesia as described [7]. Left ventricular (LV) morphology and systolic function were evaluated by two-dimensional M-mode recording by an experienced and blinded sonographer.

### Analysis for infarct size

Infarct size and the size of the area at risk (AR) following I/R were determined as previously described [1]. At the end of the 24h reperfusion period, the mice were reanaesthetized and the ligature around the LAD was retied at the previous ligation site. Following this, 2% Evans blue dye was injected into the aorta and allowed to circulate uniformly in the areas of the heart perfused by the open coronary arteries. The heart was then quickly excised, snap frozen on dry ice and cut into five sections from the apex to the base. Sections were then incubated in 1% triphenyltetrazolium chloride (TTC, Sigma-Aldrich, St. Louis, MO) solution prepared in phosphate buffered saline (PBS, Sigma-Aldrich, St. Louis, MO) for 20 min at 37°C, fixed with phosphate-buffered 10% formalin (pH 7.0) 20 min, rinsed in PBS and scanned with CanoScan 5600F (Canon, Tokyo, Japan) using MP Navigator EX software (Canon, Tokyo, Japan). The area not at risk (ANAR; Evans blue-stained area) and the AR (including both the TTC staining-positive [non-infarct, red] and TTC staining-negative [infarct, white] areas) were measured by blinded observer with the NIS-Elements BR 2.30 program (Nikon Inc., Melville, NY). Measurements for serum troponin I levels were performed according to manufacturer's instructions by using a High Sensitivity Mouse Cardiac Troponin-I ELISA Kit (#2010-1-HSP, Life Diagnostics Inc., West Chester, PA).

### Epifluorescence microscopy

For assessment of Spry1 expression in cardiac tissue, formalin fixed and paraffin-embedded tissue sections were used. Sections were incubated for 1 hour at 50°C. Paraffin was removed in xylene and the sections were dehydrated in graded ethanol series. Sections were incubated in 3% hydrogen peroxide prepared in PBS for 5 minutes at RT, to quench endogenous peroxidase activity.  Permeabilization was done with boiling in sodium citrate buffer (10 mM sodium citrate (Riedel-de Haen, Morristown, NJ), 0.05% Tween 20 (Sigma–Aldrich, St. Louis, MO), pH 6.0). Blocking buffer including 2% bovine serum albumin (BSA, VWR, Radnor, PA), 0.2% horse serum (Sigma–Aldrich, St. Louis, MO) and 0.2% Triton X-100 (Sigma–Aldrich, St. Louis, MO) in PBS was used for blocking for 1 hour at RT. Primary antibodies were diluted in blocking buffer as indicated later and incubated on tissue section overnight at 4°C. Primary antibodies used: 1:200 Sprouty1 (Spry1, 75492, Abcam, Cambridge, United Kingdom), 1:100 α-actinin (A7811, Sigma-Aldrich, St. Louis, MO), 1:100 heat shock protein 60 (HSP60, ADI-SPA-829, Enzo Life Sciences Inc., Farmingdale, NY), 1:100 phosphorylated extracellular signal-regulated kinase (p-ERK, 9106, Cell Signaling, Danvers, MA), 1:1000 Phalloidin-iFluor 488 Reagent - CytoPainter (ab176753, Abcam, Cambridge, United Kingdom). When appropriate, CY2 Conjugated Affinity Purified anti-Mouse IgG (610-711-124, Rockland, Limerick, PA), CY3 Conjugated Affinity Purified anti-Rabbit IgG (611-104-122, Rockland, Limerick, PA), or Alexa Fluor 568 (1:100, A11031, Invitrogen, Carlsbad, CA) and 1:500000 4',6-diamidino-2-phenylindole (DAPI, D9542, Sigma-Aldrich, St. Louis, MO) were diluted in blocking buffer and incubated on tissue section for 1 hour at RT. The sections were viewed with a Nikon Eclipse 80i microscope (Nikon Inc.,

Melville, NY), and captured with ORCA-Flash4.0 LT digital camera (Hamamatsu, Hamamatsu City, Japan) and NIS-Elements AR 4.30.01 software (Nikon Inc., Melville, NY).

**Fractioning of resident mouse cardiac cells**

Mouse ventricular cardiomyocytes, cardiac endothelial cells and fibroblasts were isolated from 8-week old mice as described previously [3].

**Isolation of primary rat ventricular cardiomyocytes**

Adult rat ventricular cardiomyocytes (ARVMs) were isolated from 8-12 weeks old male SD-rats by retrograde perfusion and enzymatic digestion using collagenase type 2 (Worthington Biochemical Corporation, Lakewood, NJ) as previously described [5]. Neonatal rat ventricular cardiomyocytes (NRVMs) were isolated from 2- to 4-day-old Sprague-Dawley rats (SD-rats) as described earlier [6]. ARVMs were cultured in MEM (10370-047, Gibco, Dublin, Ireland) supplemented with 0.01 % bovine serum albumin, 10 mM HEPES (Thermo Fisher Scientific, Waltham, MA), insulin-transferrin-selenium supplement (Thermo Fisher Scientific, Waltham, MA), 10 mM 2,3-butandione monoxime, 2 mM L-glutamine and 1 % penicillin-streptomycin.

**RNAi protocol**

Day after NRVM isolation, 100 nM specific Sprouty1 siRNA (Spry1 5'-GGUUAGACUAUGACAGGGAtt -3', Ambion Inc., Austin, TX) and 100 nM negative control siRNA (SIC001, Sigma-Aldrich, St. Louis, MO)) were transfected into the NRVMs using Lipofectamine™ 2000 (Invitrogen, Carlsbad, CA) as transfection reagent according to manufacturer's instructions. During transfections NRVMs were incubated in Opti-MEM® I (Gibco, Dublin, Ireland) for 24 h and thereafter the cells were incubated in serum-free medium. Exception for this are hypoxia experiments, where cells were incubated after siRNA transfection in DMEM (31885-023, Gibco, Dublin, Ireland) supplemented with 2% FBS and 1% penicillin-streptomycin (PS).

**Recombinant Adenoviral Vectors**

The pEF5/FRT/V5 GSK3β 3×FLAG and pEF5/FRT/V5 GSK3βserine9alanine 3×FLAG plasmids were a kind gift from Brad Doble. Plasmids were transformed in competent XL1Blue cells and plated on ampicillin plates. Minipreps were grown from the bacterial colonies to amplify plamids. Minipreps were purified with QIAprep Spin Miniprep Kit (27106, Qiagen, Hilden, Germany). Serine 389 mutations were induced to both plasmids to create GSK3β with serine389alanine mutation and GSK3β with both serine9alanine and serine389alanine mutations. For site-directed mutations, QuickChange II Site-Directed Mutagenesis kit (Agilent Technologies Inc., Santa Clara, CA), was used. The production of adenoviruses was performed as previously described [4]. Adenoviruses were added on NRVMs at 3 MOI on third day after siRNA transfections. 24h later, experiments were started.

**Cell death assays**

To evaluate cell death in vitro, analysis of adenylate kinase release from ruptured cells into the cell culture medium, a kit from Lonza (ToxiLight$^{TM}$, Basel, Switzerland) was used according to manufacturer's instructions. For quantification of cell death in vivo, terminal deoxynucleotidyl transferase-mediated dUTP

nick end labeling (TUNEL) was embloyed. A kit from Millipore (Merck KGaA, Darmstadt, Germany) was used according to the manufacturer's instructions. Images were viewed with a Nikon Eclipse 80i microscope (Nikon Inc., Melville, USA) and captured with ORCA-Flash4.0 LT digital camera (Hamamatsu, Hamamatsu City, Japan). NIS-Elements AR 4.30.01 software (Nikon Inc., Melville, NY) was used to record immunofluorescence images.

**Analysis for mitochondrial membrane potential**

For analysis of mitochondrial membrane potential cells were incubated with 1 µM JC-1 dye from Millipore (Merck KGaA, Darmstadt, Germany) for 30 min at +37°C. Cells were washed once with cell culture medium and hypoxia experiment was performed as described in Methods. After hypoxia fluorescent readings for JC-1 aggregate emission (590 nm) and JC-1 monomer emission (530 nm) were measured with Varioskan Flash (Thermo Scientific, Singapore, China) using Skanlt Software for Varioskan Flash version 2.4.5 (Thermo Scientific, Singapore, China).

**Detection of mitochondrial permeability transition pore opening**

For detection of mitochondrial permeability transition pore opening cells were incubated with 3 µM Calcein-AM (sc-203865, Santa Cruz Biotechnology, Inc., Dallas, TX) and 1 mM Cobalt(III) chloride ($CoCl_2$, sc-252623, Santa Cruz Biotechnology, Inc., Dallas, TX) for 30 min at +37°C. After incubation hypoxia experiment was performed as described in Methods. After hypoxia fluorescent readings for Calcein-AM emission (520 nm) were measured with Varioskan Flash (Thermo Scientific, Singapore, China) using Skanlt Software for Varioskan Flash version 2.4.5 (Thermo Scientific, Singapore, China).

**Protein isolation and immunoblotting**

Isolation of total protein and Western blotting were performed as described previously [2]. For mitochondrial protein isolation, cells from four individual experiments were pooled for each sample. The cells were lysed into homogenization buffer including 250 mM sucrose, 10 mM Tris (pH 7.4) and 1mM EDTA supplemented with 1 mM β-glycerophosphate, 1 mM $Na_3VO_4$, 1 mM 1,4-dithiothreitol (DTT), 50 mM sodium fluoride (NaF), 1:100 protease inhibitor cocktail 1 (PRO, P8340, Sigma-Aldrich, St. Louis, MO) and 1:100 phosphatase inhibitor cocktail 3 (PHO, P0044, Sigma-Aldrich, St. Louis, MO) and 1:100. After centrifugation at 1000 g, for 5 min, at +4°C, the supernatant was centrifuged again at 13 000 g, for 20 min, at +4°C to pellet the mitochondria. Solubilization buffer including 150 mM NaCl, 20 mM Tris (pH 7.4), 10 mM EDTA, 1% (v/v) Igepal supplemented with 1 mM β-glycerophosphate and 1 mM $Na_3VO_4$, 1 mM DTT, 50 mM NaF, 1:100 PRO and 1:100 PHO was used to dissolve mitochondrial protein pellets. The lysate was centrifuged at 13 000 g, for 10 min, at +4°C to remove insoluble material. Primary antibodies used were 1:1000 phosphorylated extracellular signal-regulated kinase (p-ERK, 9106, Cell Signaling, Danvers, MA), 1:2000 Sprouty1 (Spry1, 75492, Abcam, Cambridge, United Kingdom), 1:200 Sprouty2 (Spry2, AF6157, R&D Systems, Minneapolis, MN), 1:500 Sprouty4 (Spry4, pab0230-P, Covalab, Villeurbanne, France), 1:1000 phosphorylated p38 (p-p38, 9211, Cell Signaling, Danvers, MA), 1:1000 phosphorylated c-Jun N-terminal kinase (p-JNK, V7931, Promega, Madison, WI), 1:1000 phosphorylated signal transducer and activator of transcription 3 (p-STAT3, 9134, Cell Signaling, Danvers, MA), 1:1000 phosphorylated protein kinase B (p-AKT, 9217, Cell Signaling, Danvers, MA), 1:1000 phosphorylated glycogen synthase kinase-3 beta (p-GSK-3β, 9336, Cell Signaling, Danvers, MA), 1:1000 heat shock protein 60 (HSP60, ADI-SPA-829, Enzo Life Sciences Inc., Farmingdale, NY), and 1:1000000 glyceraldehyde 3-phosphate dehydrogenase

(GAPDH, MAB374, Millipore, Burlington, MA). The signals were normalized to that of GAPDH or HSP60 to correct for potential differences in loading. Quantification of Western blot analysis was performed using Image J (64-bit, NIH, Bethesda, MD).

**RNA isolation, cDNA synthesis and qRT-PCR analysis**

Total RNA isolation and qRT-PCR analyses were performed as previously described [7]. The sequences of the forward and reverse primers and the fluorogenic probes used for gene-specific cDNA detection are listed in Online Table 2. The qRT-PCR analyses were done with 7300 Real Time PCR System (Applied Biosystems, Foster City, CA) using TaqMan Chemistry (Applied Biosystems, Foster City, CA) and 7300 System SDS Software (Applied Biosystems, Foster City, CA).

## References


1. Gao E, Lei YH, Shang X, Huang ZM, Zuo L, Boucher M, Fan Q, Chuprun JK, Ma XL, Koch WJ (2010) A Novel and Efficient Model of Coronary Artery Ligation and Myocardial Infarction in the Mouse. Circulation Research 107:1445-1453 doi:10.1161/CIRCRESAHA.110.223925
2. Kaikkonen L, Magga J, Ronkainen VP, Koivisto E, Perjes A, Chuprun JK, Vinge LE, Kilpio T, Aro J, Ulvila J, Alakoski T, Bibb JA, Szokodi I, Koch WJ, Ruskoaho H, Kerkela R (2014) p38alpha regulates SERCA2a function. J.Mol.Cell Cardiol. 67:86-93 doi:10.1016/j.yjmcc.2013.12.005
3. Kerkela R, Karsikas S, Szabo Z, Serpi R, Magga J, Gao E, Alitalo K, Anisimov A, Sormunen R, Pietila I, Vainio L, Koch WJ, Kivirikko KI, Myllyharju J, Koivunen P (2013) Activation of Hypoxia Response in Endothelial Cells Contributes to Ischemic Cardioprotection. Mol.Cell Biol. 33:3321-3329 doi:10.1128/MCB.00432-13
4. Moilanen AM, Rysa J, Serpi R, Mustonen E, Szabo Z, Aro J, Napankangas J, Tenhunen O, Sutinen M, Salo T, Ruskoaho H (2012) (Pro)renin receptor triggers distinct angiotensin II-independent extracellular matrix remodeling and deterioration of cardiac function. PLoS One 7:e41404 doi:10.1371/journal.pone.0041404
5. Perjes A, Kilpio T, Ulvila J, Magga J, Alakoski T, Szabo Z, Vainio L, Halmetoja E, Vuolteenaho O, Petaja-Repo U, Szokodi I, Kerkela R (2016) Characterization of apela, a novel endogenous ligand of apelin receptor, in the adult heart. Basic Res.Cardiol. 111:1-12 doi:10.1007/s00395-015-0521-6
6. Pikkarainen S, Tokola H, Majalahti-Palviainen T, Kerkela R, Hautala N, Bhalla SS, Charron F, Nemer M, Vuolteenaho O, Ruskoaho H (2003) GATA-4 is a nuclear mediator of mechanical stretch-activated hypertrophic program. Journal of Biological Chemistry 278:23807-23816 doi:10.1074/jbc.M302719200
7. Szabo Z, Magga J, Alakoski T, Ulvila J, Piuhola J, Vainio L, Kivirikko KI, Vuolteenaho O, Ruskoaho H, Lipson KE, Signore P, Kerkela R (2014) Connective tissue growth factor inhibition attenuates left ventricular remodeling and dysfunction in pressure overload-induced heart failure. Hypertension 63:1235-1240 doi:10.1161/HYPERTENSIONAHA.114.03279

**Supplementary Tables**

**Online Table 1.**

Echocardiography analysis of left ventricular structure and function prior to cardiac ischemia.

| | **WT** (n=6) | **Spry1$^{fl/fl}$** (n=6) | **Spry1 cKO** (n=6) |
|---|---|---|---|
| **LVID;d (mm)** | 3.98±0.18 | 4.04±0.20 | 4.07±0.13 |
| **LVID;s (mm)** | 2.85±0.23 | 2.89±0.15 | 2.89±0.19 |
| **LVPW;d (mm)** | 0.80±0.07 | 0.77±0.04 | 0.76±0.03 |
| **LVPW;s (mm)** | 1.12±0.07 | 1.12±0.07 | 1.04±0.07 |
| **LVVol;d (µl)** | 69.46±7.21 | 74.52±9.27 | 74.56±3.22 |
| **LVVol;s (µl)** | 31.34±6.29 | 31.78±4.52 | 30.47±5.76 |
| **EF (%)** | 57.44±6.22 | 57.41±2.01 | 59.36±5.03 |
| **FS (%)** | 29.92±4.08 | 29.82±1.32 | 31.24±3.47 |
| **HR (BPM)** | 431±29 | 433±31 | 435±49 |
| **SV (µl)** | 40.90±4.27 | 42.74±5.16 | 44.10±3.22 |
| **CO (ml/min)** | 17.64±2.22 | 18.53±2.70 | 19.20±2.80 |

Cardiac structure and function were analyzed by echocardiography. LVID;d = LV end-diastolic dimension; LVID;s = LV end-systolic dimension; LVPW;d = end-diastolic posterior wall thickness; LVVol;d = LV end-diastolic volume; LVVol;s = LV end-systolic volume; EF = ejection fraction; FS = fractional shortening; HR = heart rate; SV = stroke volume; CO = cardiac output; Data is presented as mean±SD.

**Online Table 2.**
The sequences of the forward (F) and reverse (R) primers and the fluorogenic probes (P) used for quantitative real-time PCR.

| Gene | Primers and Probes |
|---|---|
| Mouse SPRY1 | (F) GCCGGCAGAGGTTAGACTATGA<br>(R) GCTTTGATCTGGTCTAGGGACAGA<br>(P) Fam-AGGGACACTCAGCCTGCTACG-Tam |
| Rat SPRY1 | (F) GCCGGCAGAGGTTAGACTATGA<br>(R) GCTTTGATCTGGTCTAGGGACAGA<br>(P) Fam-AGGGACACCCAGCCTGCCACG-Tam |
| Rat GAPDH | (F) GGTCATCATCTCCGCCCC<br>(R) TTCTCGTGGTTCACACCCATC<br>(P) Fam-CTGCCGATGCCCCCATGTTTG-Tam |
| Mouse/Rat18S | (F) TGGTTGCAAAGCTGAAACTTAAAG<br>(R) AGTCAAATTAAGCCGCAGGC<br>(P) Fam-CCTGGTGGTGCCCTTCCGTCA-Tam |
| Mouse αMHC | (F)GGTGCCAAGAAGATGCACG<br>(R) TTATGTTTATTGTGTATTGGCCACAG<br>(P) Fam-CGAGGAATAACCTCTCCAGCAGACCCTC-Tam |
| Mouse Col1a1 | (F)CCCTGGCCTTGGAGGAA<br>(R) CACGGAAACTCCAGCTGATTTT<br>(P) Fam- CTTTGCTTCCCAGATGTCCTATGGCTATGATG –Tam |
